# Supplementary material for: T-Cell Responses to COVID-19 Vaccines and Breakthrough Infection in People Living with HIV Receiving Antiretroviral Therapy
Source: Viruses. 2024 Apr 24;16(5):661. doi: 10.3390/v16050661 (PMC11125792; doi:10.3390/v16050661)
Supplement: Supplementary file 1 [file viruses-16-00661-s001.zip › viruses-2949056-supplementary.pdf]

Table S1. Multivariable zero-inflated beta regressions exploring the relationship between sociodemographic, health and vaccine-related variables on Spike-specific CD4+ T cell responses following two and three COVID-19 vaccine doses.

Variable	Outcome measures					
	% Spike-responsive CD4+ T cells after two vaccine doses			% Spike-responsive CD4+ T cells after three vaccine doses		
	**Estimate**	**Standard error**	**P value**	**Estimate**	**Standard error**	**P value**
PLWH group	-0.29	0.17	0.08	-0.06	0.10	0.58
Age (per year)	0.001	0.004	0.83	-0.004	0.002	0.08
Male sex	0.13	0.14	0.38	0.24	0.084	**0.0044**
White ethnicity	-0.19	0.14	0.16	-0.045	0.076	0.55
Health conditions (per condition)[a]	-0.03	0.08	0.72	0.11	0.047	**0.022**
ChAdOx1-containing initial vaccine regimen[b]	0.37	0.21	0.09	0.14	0.12	0.27
Days between 1st and 2nd vaccine dose (per day)	0.005	0.003	0.12	0.002	0.002	0.30
mRNA-1273 as third vaccine dose[c]	N.A.	N.A.	N.A.	0.17	0.077	**0.033**
Days between 2nd and 3rd vaccine dose (per day)	N.A.	N.A.	N.A.	0.001	0.001	0.47
% Spike specific T cells after two doses (per 1% increment)	N.A.	N.A.	N.A.	0.71	0.058	**$<2x10^{-16}$**

[a]Continuous variable, defined as: chronic blood disorder, cancer, hypertension, diabetes, asthma, obesity (body mass index ≥30), chronic diseases of lung, liver, kidney or heart. [b]Binary variable, defined as two doses of ChAdOx1, or a heterologous regimen consisting of one dose each of ChAdOx1 and an mRNA vaccine (mRNA-1273 or BNT162b2) (vs. a reference group defined as two doses of mRNA vaccine). [c]Binary variable, defined as a third dose of mRNA-1273 (vs. a reference group defined as a third dose of BNT162b2).

Table S2. Multivariable zero-inflated beta regressions exploring the relationship between sociodemographic, health and vaccine-related variables on Spike-specific CD8+ T cell responses following two and three COVID-19 vaccine doses.

Variable	Outcome measures					
	% Spike-responsive CD8+ T cells after two vaccine doses			% Spike-responsive CD8+ T cells after three vaccine doses		
	**Estimate**	**Standard error**	**P value**	**Estimate**	**Standard error**	**P value**
PLWH group	-0.0002	0.16	0.99	-0.24	0.12	**0.045**
Age (per year)	-0.001	0.004	0.87	0.001	0.004	0.87
Male sex	0.18	0.14	0.20	0.11	0.10	0.24
White ethnicity	0.14	0.13	0.30	-0.01	0.08	0.90
Health conditions (per condition)[a]	-0.02	0.08	0.75	-0.05	0.06	0.37
ChAdOx1-containing initial vaccine regimen[b]	0.39	0.20	**0.049**	0.22	0.14	0.12
Days between 1st and 2nd vaccine dose (per day)	0.002	0.003	0.54	0.00	0.00	0.16
mRNA-1273 as third vaccine dose[c]	N.A.	N.A.	N.A.	0.02	0.11	0.86
Days between 2nd and 3rd vaccine dose (per day)	N.A.	N.A.	N.A.	-0.001	0.002	0.43
% Spike specific T cells after two doses (per 1% increment)	N.A.	N.A.	N.A.	0.79	0.07	**<2e-16**

[a]Continuous variable, defined as: chronic blood disorder, cancer, hypertension, diabetes, asthma, obesity (body mass index ≥30), chronic diseases of lung, liver, kidney or heart. [b]Binary variable, defined as two doses of ChAdOx1, or a heterologous regimen consisting of one dose each of ChAdOx1 and an mRNA vaccine (mRNA-1273 or BNT162b2) (vs. a reference group defined as two doses of mRNA vaccine). [c]Binary variable, defined as a third dose of mRNA-1273 (vs. a reference group defined as a third dose of BNT162b2).

Table S3. Multivariable logistic regression looking at factors associated with risk of first breakthrough infection between 1 and 6 months following receipt of the 3rd COVID-19 vaccine dose.

Variables	Estimate	95% CI	P value
PLWH group	0.93	0.30 to 2.78	0.89
Age (per year)	0.96	0.93 to 0.98	**0.002**
Male sex	0.68	0.27 to 1.66	0.40
White ethnicity	1.55	0.69 to 3.60	0.29
Health conditions (per condition)[a]	1.22	0.72 to 2.09	0.46
ChAdOx1-containing initial vaccine regimen[b]	2.02	0.52 to 8.27	0.31

Days between 1st and 2nd vaccine dose (per day)	1.00	0.97 to 1.02	0.76
mRNA-1273 as third vaccine dose[c]	1.09	0.49 to 2.47	0.84
Days between 2nd and 3rd vaccine dose (per day)	1.00	0.99 to 1.01	0.96
% Spike specific CD4+ T cells after three doses (per 1% increment)	0.76	0.25 to 2.13	0.61
% Spike specific CD8+ T cells after two doses (per 1% increment)	0.86	0.36 to 2.01	0.73
Virus neutralizing activirty (per 1% increment)	1.00	0.99 to 1.00	0.26

[a]Continuous variable, defined as: chronic blood disorder, cancer, hypertension, diabetes, asthma, obesity (body mass index ≥30), chronic diseases of lung, liver, kidney or heart. [b]Binary variable, defined as two doses of ChAdOx1, or a heterologous regimen consisting of one dose each of ChAdOx1 and an mRNA vaccine (mRNA-1273 or BNT162b2) (vs. a reference group defined as two doses of mRNA vaccine). [c]Binary variable, defined as a third dose of mRNA-1273 (vs. a reference group defined as a third dose of BNT162b2).

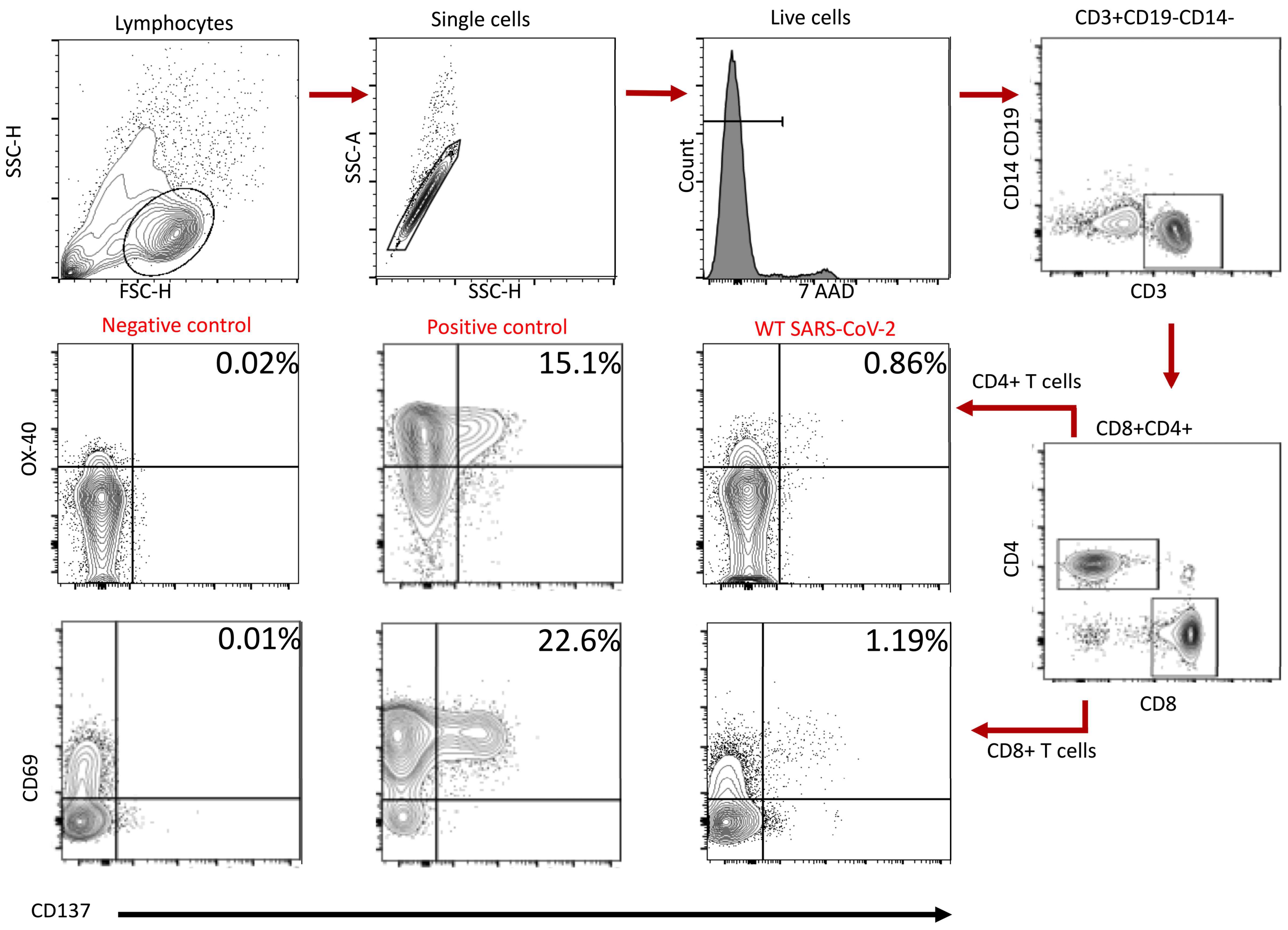

Figure S1. Gating strategy for quantification of SARS-CoV-2 spike-specific CD4+ and CD9+ T cells using an activation induced marker (AIM) assay.

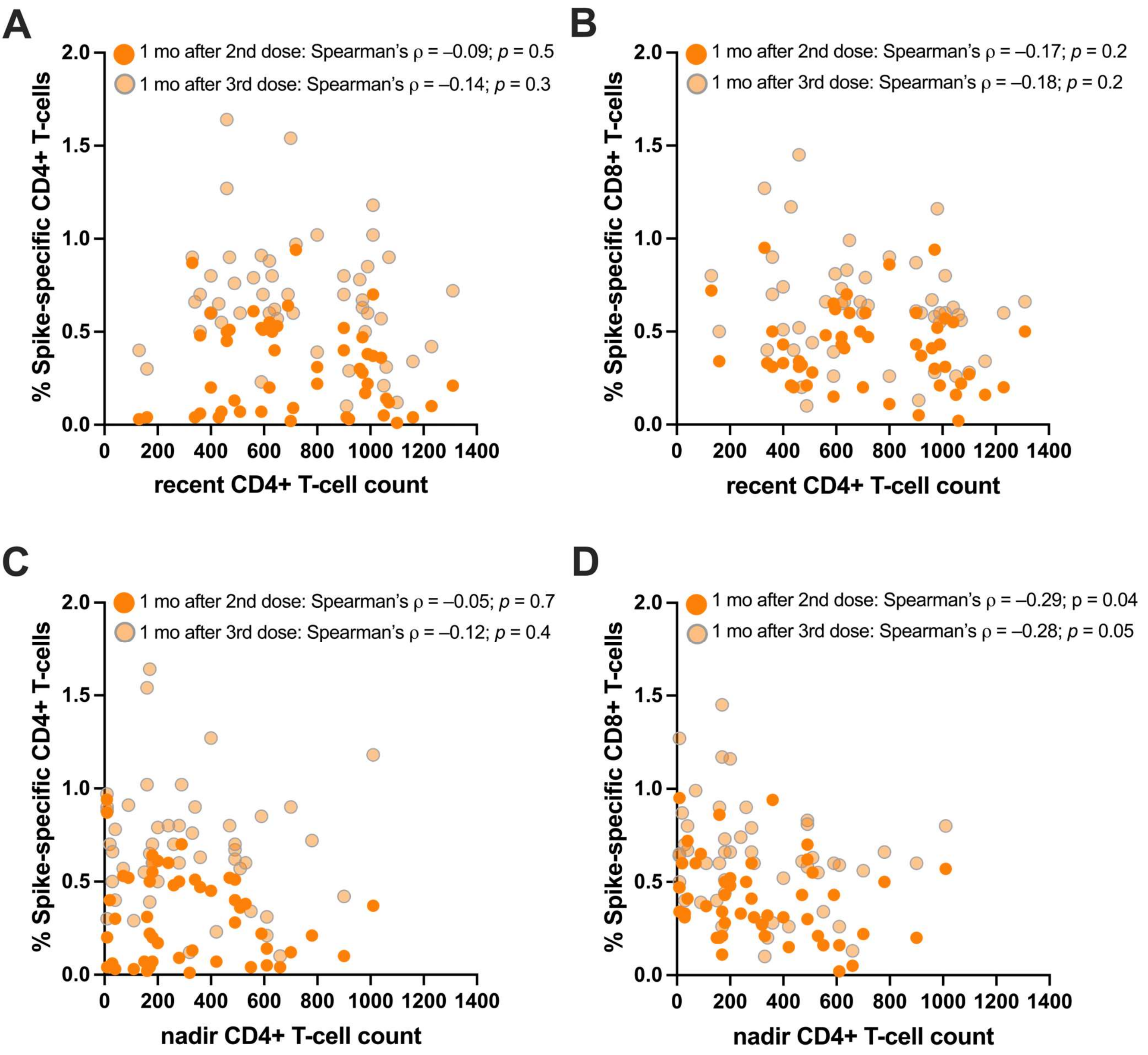

Figure S2. Correlations between vaccine-induced spike-specific T cell responses and recent or nadir CD4+ T cell count in PLWH.
